# Novel Nonstructural Protein 4 Genetic Group in Rotavirus of Porcine Origin

**DOI:** 10.3201/eid1404.07111

**Published:** 2008-04

**Authors:** Pattara Khamrin, Shoko Okitsu, Hiroshi Ushijima, Niwat Maneekarn

**Affiliations:** * The University of Tokyo, Tokyo, Japan; † Aino University, Tokyo, Japan; ‡ Chiang Mai University, Chiang Mai, Thailand

**Keywords:** Rotavirus, NSP4 genetic group, porcine, letter

## Abstract

Novel Genetic Group in Rotavirus

**To the Editor:** Infection with group A rotavirus is the main cause of acute gastroenteritis in infants and young children worldwide and in young animals of many species, including piglets. In recent years, several epidemiologic studies designed to monitor the appearance of novel or atypical rotavirus antigenic types have provided evidence for the increasing antigenic diversity of group A rotaviruses (*1*–*3*). In addition to the 2 rotavirus classification systems, VP7 (G) and VP4 (P) genes, the virus can also be classified on the basis of the nonstructural glycoprotein 4 (NSP4)–encoding gene. Sequence analyses of the NSP4 gene indicated the presence of at least 5 distinct genetic groups among human and animal rotaviruses, termed A to E (*1*,*4*,*5*). Among human rotaviruses, the diversity of NSP4 genes has been restricted mainly to genetic groups A and B; only a few human strains possess genetic group C. Conversely, all 5 NSP4 genetic groups (A–E) have been identified in rotaviruses of animal origins. To our knowledge, porcine rotaviruses (PoRVs) have been reported to belong only to NSP4 genetic group B (*1*).

During an epidemiologic survey of PoRV from June 2000 through July 2001, a total of 175 fecal specimens were collected from diarrheic piglets from 6 different farms in Chiang Mai Province, Thailand. Of these, 39 (22.3%) specimens were positive for group A rotavirus (*6*). A novel and unusual PoRV CMP034 strain was isolated from a 7-week-old piglet during this survey. Molecular genetic characterization showed that the CMP034 strain carried a novel P[27] genotype with a new lineage of G2-like rotavirus genotype (*7*). We performed a molecular analysis of the NSP4 gene of this strain in comparison with those of other NSP4 gene sequences available in the GenBank database.

The full-length of NSP4 gene was amplified by NSP4–1a and NSP4–2b primer pairs (*8*). The PCR amplicon was sequenced in both directions by using the BigDye Terminator Cycle Sequencing kit (PerkinElmer-Applied Biosystems, Inc., Foster City, CA, USA) on an automated sequencer (ABI 3100; PerkinElmer-Applied Biosystems, Inc.). The sequence of CMP034 was compared with those of reference strains available in the National Center for Biotechnology Information GenBank database by using BLAST (www.ncbi.nlm.nih.gov/blast). The NSP4 nucleotide sequence of the CMP034 strain was deposited in GenBank under accession no. DQ534017.

The complete NSP4 nucleotide sequence of PoRV CMP034 strain was 750 bp and contained a single long open reading frame coding for a protein of 175 aa. Comparative analysis of the CMP034 NSP4 sequence with those of the 5 representative established genetic groups (A–E) showed the highest sequence identity, at 92.6% nt and 96.9% aa levels, with 1 PoRV strain, P21–5 (*9*). However, CMP034 and P21–5 shared a low degree of sequence identity with other NSP4 genetic groups. The NSP4 sequence identities of the CMPO34 and P21-5 strains ranged from 74% to 78% nt and 75%–79% aa levels with those of genetic group A; 77%–86% nt and 79%–86% aa levels with genetic group B; 69%–73% nt and 75%–78% aa levels with genetic group C; 62%–65% nt and 55%–60% aa levels with genetic group D; and only 43%–50% nt and 29%–33% aa levels with genetic group E. The phylogenetic tree confirmed that PoRV strains CMP034 and P21–5 were located exclusively in a separated branch, which was distantly related to the other 5 known NSP4 genetic groups (Figure). However, a bootstrap support for the separation of the gene into a separate lineage is very strong with nucleotide sequencing but weak by amino acid analysis in this phylogentic tree. Our finding indicates that PoRV strains CMP034 and P21–5 are likely a novel NSP4 genetic group and, therefore, tentatively proposed as a NSP4 genetic group F.

**Figure:**
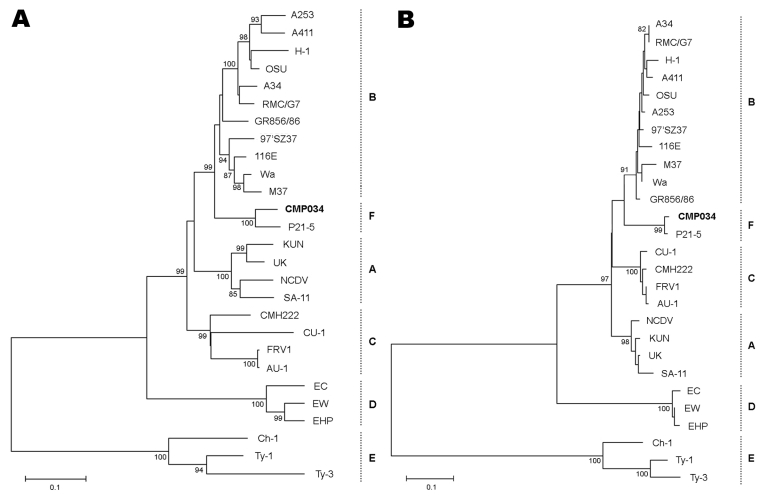
Phylogenetic analyses of the NSP4 nucleotide (A) and amino acid (B) sequences displaying the relationships between porcine rotavirus strain CMP034 (shown in **boldface**), P21–5, and other 5 known NSP4 genetic groups. Bootstrap values are shown at the branch nodes. Branch length for a 10% nucleotide difference is indicated at the bottom.

On the basis of the accumulated evidence of transmission of rotaviruses between pigs and other animal species, including humans, pigs are regarded as 1 potential reservoir for the emergence of unusual or novel strains of rotaviruses (*6*,*7*). In our study, the virus carried a novel NSP4 genetic group that has been isolated from a diarrheic piglet in Thailand. The NSP4 sequence analysis of our CMP034 strain revealed a PoRV strain closely related genetically to the NSP4 gene sequence of PoRV strain P21–5 isolated in Slovenia (*9*). PoRV strains CMP034 and P21–5 shared the same VP4 genotype as P[27] with over 90% aa sequence identity. The only difference observed between the 2 strains was that CMP034 belonged to the G2-like genotype whereas P21–5 belonged to G1 genotype. The relatedness between NSP4 sequences of strains CMP034 and P21–5 was confirmed by phylogenetic analysis, which showed that both CMP034 and P21–5 clustered closely together in a branch separated from those of other 5 NSP4 genetic groups. This finding suggests that NSP4 of PoRV strain CMP034 and P21–5 may have derived from the same ancestor. The isolation of 2 strains of rotaviruses with a close genetic relatedness of NSP4 gene from Thailand and Slovenia, 2 countries that are located in different continents, may indicate that this novel NSP4 genetic group has already been introduced into PoRVs worldwide. To verify this hypothesis, extensive epidemiologic surveillance of rotavirus in pigs may need to be conducted in several other regions of the world.
